# *Pneumocystis jirovecii* pneumonia in liver transplant recipients in an era of routine prophylaxis

**DOI:** 10.1002/iid3.546

**Published:** 2021-10-29

**Authors:** Philip B. Andreasen, Omid Rezahosseini, Dina L. Møller, Neval E. Wareham, Magda T. Thomsen, Ranya Houmami, Andreas D. Knudsen, Jenny Knudsen, Jørgen A. L. Kurtzhals, Andreas A. Rostved, Christian R. Pedersen, Allan Rasmussen, Susanne D. Nielsen

**Affiliations:** ^1^ Viro‐immunology Research Unit, Department of Infectious Diseases 8632 Copenhagen University Hospital, Rigshospitalet Copenhagen Denmark; ^2^ Centre of Excellence for Personalized Medicine of Infectious Complications in Immune Deficiency Rigshospitalet Copenhagen Denmark; ^3^ Department of Cardiology Copenhagen University Hospital, Rigshospitalet Copenhagen Denmark; ^4^ Department of Clinical Microbiology Copenhagen University Hospital, Rigshospitalet Copenhagen Denmark; ^5^ Centre for Medical Parasitology, Department of Immunology and Microbiology University of Copenhagen Copenhagen Denmark; ^6^ Department of Surgical Gastroenterology and Transplantation, Rigshospitalet University of Copenhagen Copenhagen Denmark; ^7^ Department of Clinical Medicine University of Copenhagen Copenhagen Denmark

**Keywords:** incidence, liver transplantation, *Pneumocystis jirovecii* pneumonia, prophylaxis, trimethoprim sulfamethoxazole

## Abstract

**Background:**

*Pneumocystis jirovecii* pneumonia (PCP) is an opportunistic infection in organ transplant recipients that may be prevented by antibiotic prophylaxis. We aimed to investigate the incidence rate (IR) of PCP and the related hospitalization and mortality rates in liver transplant recipients in an era of routine prophylaxis.

**Methods:**

We included all adult liver transplant recipients transplanted at Rigshospitalet between January 1, 2011 and October 1, 2019. Microbiology data were obtained from the Danish Microbiology Database (MiBa), a national database containing all data from all Departments of Clinical Microbiology in Denmark receiving samples from both hospitals and general practices. According to local guidelines, PCP prophylaxis was initiated 1 week posttransplantation and discontinued after 6 months or sooner in patients experiencing side effects.

**Results:**

We included 343 liver transplant recipients with 1153 person‐years of follow‐up (PYFU), of which 269 (78%) received PCP prophylaxis during the first 6 months posttransplantation. Seven (2%) recipients were diagnosed with PCP during follow‐up. In the first 6 months posttransplantation and in 269 transplant recipients who received prophylaxis there were zero PCP events while the IR was 32 (95% confidence interval [CI] 2.9–148) per 1000 PYFU in 74 recipient who did not receive prophylaxis. During 7th to 12th month posttransplantation the IR was 20 (95% CI: 5.5–53) per 1000 PYFU. All seven (100%) recipients diagnosed with PCP were hospitalized, however none died.

**Conclusions:**

PCP was not detected in liver transplant recipients while on prophylaxis. Though, it worth mentioning that two out of the seven PCP patients received high‐dose prednisolone before the PCP event. All liver transplant recipients with PCP were hospitalized, but none died. Randomized clinical trials to determine the optimal duration of prophylaxis are warranted.

## Graphical abstract

We investigated the incidence of *Pneumocystis jirovecii* pneumonia (PCP) in a cohort of liver transplant recipients in an era with the use of routine PCP prophylaxis. There were no cases of PCP in recipients while on prophylaxis. Furthermore, no case with PCP infection was reported after 15th month posttransplantation. This underlines the need for randomized clinical trials to determine the optimal duration of prophylaxis.

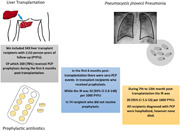

## 1 INTRODUCTION

Solid organ transplantation (SOT) is a life‐saving treatment for patients with end‐stage organ failure, and SOT increases both the survival and quality of life of the recipient.^1^ Posttransplantation, most SOT recipients receive life‐long immunosuppressive therapy to prevent rejection of the transplanted organ. However, the use of immunosuppressive therapy increases the risk of infections, including opportunistic infections.^2^

*Pneumocystis jirovecii* is an opportunistic fungal pathogen that mainly infects immunocompromised individuals, including SOT recipients.^3^ The fungus has affinity to the lung tissue, in particular Type I alveolar epithelium. Host immune and inflammatory responses to the fungus results in lung injury and *P. jirovecii* pneumonia (PCP). Fever, dyspnea, and cough are the most common clinical findings in SOT recipients with PCP.^3^ However, PCP may have a complicated course in SOT recipients with as many as 49% needing intensive care unit (ICU) admission and a mortality of 39%.^4^ Use of routine PCP prophylaxis has been shown to decrease the incidence of PCP in SOT recipients.^5^, ^6^, ^7^ Hence, the use of prophylaxis is recommended in part of the transplantation centers worldwide with trimethoprim‐sulfamethoxazole (TMP‐SMZ) being the drug of choice.^3^ However, despite the routine use of prophylaxis, SOT recipients with PCP have significant risk of hospitalization and mortality.^6^ Previous studies in SOT recipients have reported an incidence rate of 3.7 per 1000 person‐years of follow‐up (PYFU) in Switzerland,^6^ and 3.0 per 1000 PYFU in France in cohorts where PCP prophylaxis was used routinely.^8^ The incidence rate was 0.2 to 10 per 1000 PYFU in liver‐transplant recipients.^5^

At Rigshospitalet, the largest Danish transplantation center, an outbreak of PCP among kidney and liver transplant recipients was reported in the years 2007–2010.^9^ During this period, PCP prophylaxis was not routinely used.^9^ Following this outbreak, the routine use of PCP prophylaxis was implemented in 2011 for both kidney and liver transplant recipients. In this study, we aimed to determine the incidence rate and the related hospitalization, ICU‐admission, and 180‐days all‐cause mortality in liver transplant after the implementation of routine PCP prophylaxis.

## 2 MATERIALS AND METHODS

### 2.1 Study design and population

In this cohort study, we present data from the Knowledge Center for Transplantation database at Rigshospitalet, Copenhagen University Hospital. The database is part of an ongoing project which aims to provide information about complications after SOT. At present, the Knowledge Center for Transplantation database contains information on all Danish adult liver transplant recipients, and we included all adult (≥18 years) liver transplant recipients who were transplanted between January 1, 2011 and October 1, 2019.

Liver transplant recipients were followed from the date of transplantation to first positive *P. jirovecii* infection, retransplantation, death, end of 5th year (day 1826) posttransplantation, or end of follow‐up on October 1, 2020 whichever came first.

In Denmark, all citizens have a unique personal civil registration number. All data were collected from patient records retrospectively using the patients' civil registration number. The Knowledge Center for Transplantation database contains pretransplantation variables such as age at transplantation, sex, comorbidities, and date of transplantation, and posttransplantation variables including acute graft rejections, retransplantation, microbiology results, and use of prophylactic antibiotics. Outcome variables of relevance to PCP were hospitalization, PCP treatment, pneumonia, ICU admission, mechanical ventilation, and death. Microbiology data were obtained from the Danish Microbiology Database (MiBa), a national database containing all data from all Departments of Clinical Microbiology in Denmark receiving samples from both hospitals and general practices with, complete coverage since 2010.^10^

Retrieval of data was approved by the Center for Regional Development (R‐20051155). According to Danish legislation, no further approval was needed.

### 2.2 Definitions

We defined *P. jirovecii* infection as a positive immunofluorescence microscopy or a positive polymerase chain reaction (PCR) on bronchial alveolar lavage (BAL), tracheal secretion, sputum (spontaneous or induced), transbronchial or open lung biopsy or a positive PCR on mouth wash.

### 2.3 Outcome definitions

Pneumonia was defined as a chest X‐ray image or computed tomography (CT)‐scan with infiltrates consistent with pneumonia during the period 7 days before and up to 30 days after detection of *P. jirovecii* infection. ICU admission and mechanical ventilation during this period were assumed related to *P. jirovecii* infection if less than 7 days before or up to 30 days after detection of *P. jirovecii* infection. All‐cause mortality (mortality) was defined as death within 180 days of *P. jirovecii* infection. Rejection was defined as a liver biopsy with evidence of acute graft rejection when required treatment with 1 g of methylprednisolone for 3–5 days.

### 2.4 Laboratory diagnosis of *P. jirovecii* infection

PCR testing was performed as part of routine clinical care. *P. jirovecii* DNA was detected using a touch down PCR method (from 2011) or an in‐house, quantitative real‐time PCR (from 2014) as described previously.^11^, ^12^ Confirmatory immunofluorescence microscopy on BAL was performed when possible in case of a positive PCR result.^11^, ^12^

Immunofluorescence microscopy on BAL‐fluid after a dithiothreitol‐concentration step was considered the gold standard diagnostic method and was performed using the MONOFLUO^TM^ *Pneumocystis jirovecii* immunofluorescence assay kit (BIO‐RAD #32515), according to the manufacturer's instruction.^12^

### 2.5 Immunosuppressive regimes and antibiotic prophylaxis

We used a standard protocol for immunosuppression: Following liver transplantation, a single dose of methylprednisolone 1000 mg was given intraoperatively. Following the transplantation, prednisolone was tapered gradually from 200 mg on Day 1 to 30 mg on Day 5. For the remaining first month, 20 mg was given daily, Months 1–2 15 mg was given daily, Months 2–3 10 mg was given daily, Months 3–6 7.5 mg was given daily. For the next 6 months, 5 mg was given daily after which the drug normally was discontinued. Tacrolimus was dosed aiming at through levels of 10–12 ng/ml in the first month, 8–10 ng/ml in Month 2–3, 7–9 ng/ml in Months 3–6, 6–8 ng/ml in Months 6–12, and 4–6 ng/ml after 1 year. Mycophenolate mofetil was given twice daily at a dosage of 1000 mg during the entire period. In liver–kidney transplant recipients and recipients with impaired kidney function, induction therapy was basiliximab 20 mg on Day 0 and 4 with delayed initiation of tacrolimus.^13^ Immunosuppression was tapered in patients with confirmed PCP infection.

PCP prophylaxis consisted of TMP‐SMZ, equivalent to 80/400 mg daily. According to local and international guidelines, PCP prophylaxis was initiated 1 week posttransplantation and discontinued after 6 months or sooner in patients experiencing side effects.^3^ The reason for not receiving or stopping prophylaxis was retrieved from patient's journals.

### 2.6 Statistical analysis

We reported proportions as frequencies (percentage), and continuous data as medians with interquartile ranges (IQR). We used Mann–Whitney *U* test to compare the differences in medians, and *χ* ^2^ test to test the frequency distributions.

Number of cases and incidence rate of PCP were reported for the first 5 years of follow‐up. Furthermore, we reported the incidence rates for the first 6 months posttransplantation (in transplant recipients who received and did not receive PCP prophylaxis) and the Months 7–12 posttransplantation (after discontinuation of PCP prophylaxis). The incidence rate was calculated as the number of recipients with PCP per PYFU. Estimates of the cumulative incidence of PCP were calculated using the Aalen‐Johansen estimator with death and retransplantation as competing risks. We calculated 95% confidence intervals (CI) using Byar's approximation to the Poisson distribution. We performed Gray's test to investigate the statistical differences in cumulative incidence. Risk factors for PCP were investigated in uni‐ and multivariable Cox proportional hazards model with rejection as time‐dependent covariate. The model was adjusted for age and sex. All analyses were conducted in the statistical software R version 3.6.1. *p* ≤ .05 were considered statistically significant.^14^

## 3 RESULTS

### 3.1 Patient characteristics

We Included 343 adult first‐time liver transplant recipients. The median age at the time of transplantation was 50 years (IQR: 42–57), and 201 (59%) participants were male. Among 343 liver transplant recipients, 269 (78%) received PCP prophylaxis. The reason for not receiving prophylaxis was not mentioned in most of the cases. Though, allergic reactions to TMP‐SMX (two patients), liver toxicity (one patient), bone marrow suppression (one patient), and renal insufficiency (one patient) were reported.

Seven (2%) of the 343 liver transplant recipients were diagnosed with PCP. There were zero cases in recipients who received PCP prophylaxis during the first 6 months posttransplantation (while on prophylaxis). One liver transplant recipient did not receive PCP prophylaxis due to bone marrow suppression and had PCP within the first 6 months posttransplantation. Six liver transplant recipients developed PCP after the 6th month (after discontinuation of PCP prophylaxis). Three during 7th to 12th months and three others during 13th to 15th months. Patient characteristics are shown in Table 1. None of the seven PCP patients received secondary PCP prophylaxis and none of them had a second episode of PCP infection during follow ups.

**Table 1:** Characteristics of the liver transplant recipients who had and did not have *Pneumocystis jirovecii* pneumonia (PCP)

Characteristics	PCP (*n* = 7)	No PCP (*n* = 336)	Total (*n *= 343)
Age (years, median (IQR))	54.4 [45.6, 61.7]	49.7 [41.6, 57.2]	49.8 (41.6, 57.2)
Male (*n*, %)	3 (43)	198 (59)	201 (59)
Rejection (*n*, %)	3 (43)	95 (28)	98 (28.6)
PCP prophylaxis during the first 6 months posttransplantation (*n*, %)	6 (86)	254 (76)	260 (76)

Abbreviation: IQR, interquartile range.

### 3.2 Incidence of PCP

In the total follow‐up of 1153 person‐years and with a median follow‐up of 3.4 years per liver transplant recipients, the IR of PCP in the first 5 years posttransplantation was 6.1 (95% CI: 2.7–12) per 1000 PYFU. We had no PCP in the first 6 months posttransplantation among 269 liver transplant recipients who received PCP prophylaxis. The IR in the first 6 months posttransplantation among liver transplant recipients who did not receive PCP prophylaxis (74 patients) was 32 (95% CI: 2.9–148) per 1000 PYFU. From 7th to 12th months posttransplantation (after discontinuation of PCP prophylaxis) the IR was 20 (95% CI: 5.5–53) per 1000 PYFU.

**Figure 1:**
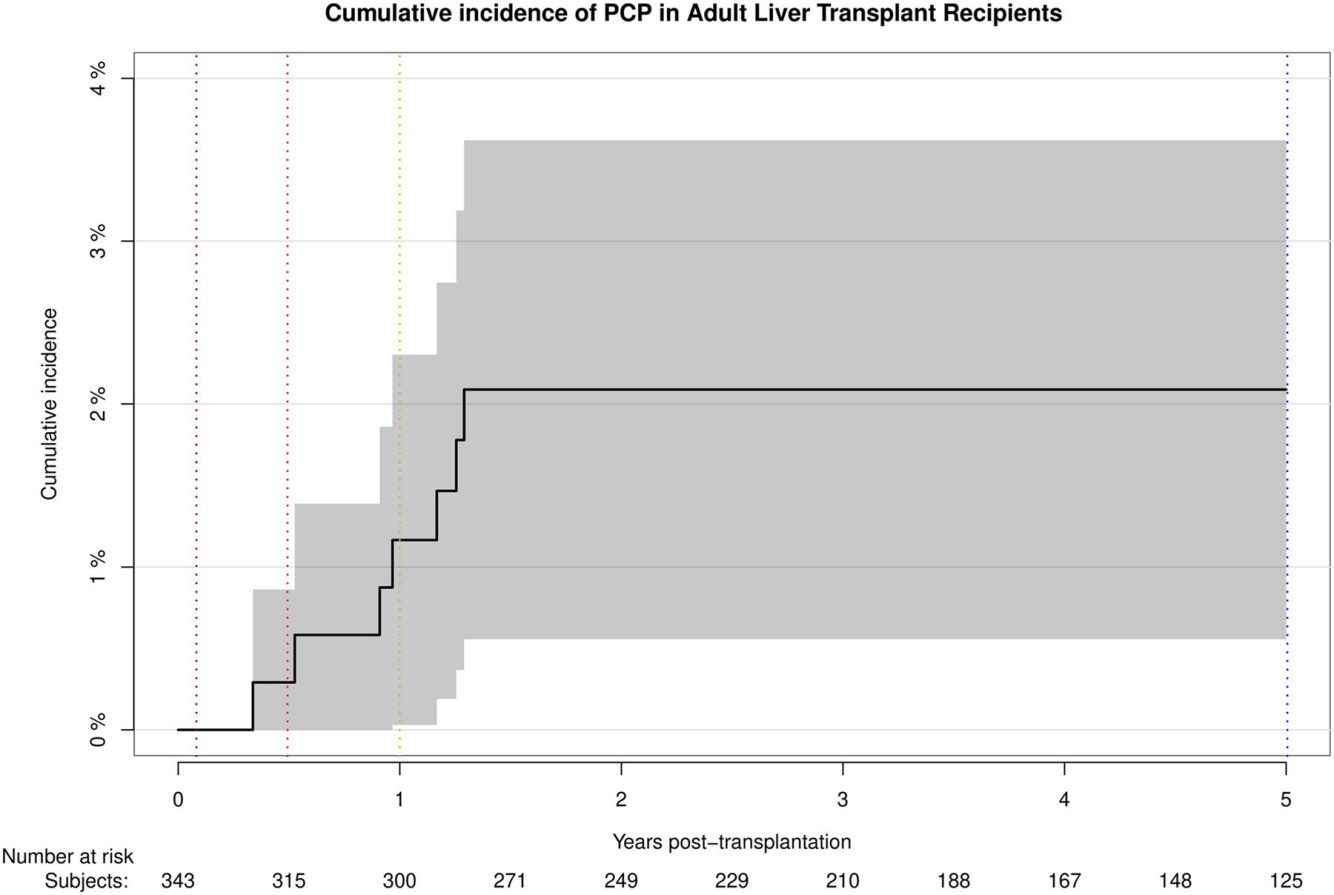
Cumulative incidence of the *Pneumocystis jirovecii* pneumonia (PCP) during the first 5 years after liver transplantation. The cumulative incidence of PCP 5 years after transplantation was 2.1% (95% CI: 0.56–3.6) and there were no cases of PCP later than 15 months posttransplantation. Vertical dotted‐lines determine 30 days (brown), 6 months (red), 1 year (yellow) and 5‐years (blue) posttransplantation. CI, confidence interval

The cumulative incidence of PCP 5 years after transplantation was 2.1% (95% CI: 0.56–3.6) (Figure 1) and there were no cases of PCP later than 15 months posttransplantation.

### 3.3 Treatment and clinical outcomes of PCP

The most common treatment for PCP was high‐dose TMP‐SMZ which was prescribed to five (71%) of the seven liver transplant recipients who had PCP. One of these later had the treatment changed to a combination of clindamycin and primaquine due to side effects. First‐line PCP treatment in one liver transplant recipient was clindamycin and primaquine due to renal insufficiency (Table 2).

**Table 2:** Characteristics, clinical outcomes, and treatment‐related variables in seven liver transplant recipients who had *Pneumocystis jirovecii* pneumonia (PCP)

Variable	The SOT recipient's number						
	1	2	3	4	5	6^a^	7
Sex	Male	Male	Female	Female	Female	Female	Male
Age (at transplantation, years)	44	64	54	47	59	64	34
PCP prophylaxis during the first 6 months posttransplantation (*n*, %)	No	Yes	Yes	Yes	Yes	Yes	Yes
Time of infection since transplantation (days)	123	458	192	332	471	353	426
Diagnostic method^e^	Microscopy of BAL	Microscopy of BAL	Microscopy of BAL	PCR on BAL	PCR on sputum	Microscopy of lung biopsy	PCR on BAL
Treatment of PCP	TMP‐SMZ + Prednisolone	TMP‐SMZ + Prednisolone	TMP‐SMZ + Prednisolone	TMP‐SMZ, clindamycin + primaquine^b^ + Prednisolone	TMP‐SMZ	TMP‐SMZ	Clindamycin + primaquine
Rejection (number of days before or after PCP)	Yes (−103)	No	Yes (−184, −71)	No	No	Yes (228)	No
Pulse‐steroid therapy for Rejection	Large standard methyl‐prednisolone^c^	−	Large standard methyl‐prednisolone^c^	−	−	Small standard methyl‐prednisolone^d^	−
Hospital admission	Yes	Yes	Yes	Yes	Yes	Yes	Yes
Verified pneumonia (On X‐ray image or CT‐scan)	Yes	Yes	Yes	Yes	No (a normal X‐ray and CT‐scan)	Yes (On CT‐scan)	Yes (On CT‐scan)

Abbreviations: BAL, bronchial alveolar lavage; CT‐scan, computerized tomography; PCP, *P. jirovecii* pneumonia; PCR, polymerase chain reaction; SOT, solid organ transplantation; TMP‐SMZ, trimethoprim‐sulfamethoxazole.

^a^ This patient was lobectomized due to lung tumor.

^b^ Patient was initially treated with TMP‐SMZ but later switched to clindamycin + primaquine.

^c^ One gram of solumedrol for 5 days followed by recycling.

^d^ One gram of solumedrol for 3 days followed by recycling.

^e^ Microscopy was performed using Immunofluorescence.

Three (43%) of seven liver transplant recipients with PCP had at least one episode of graft rejection. Two of these had rejection episodes 71–184 days before the PCP diagnosis. One of the two had a single episode of rejection 103 days before his PCP infection and was treated with pulse‐steroid (1 g of solumedrol) for 5 days, while the other patient had two episodes of graft rejection, 184 and 71 days before the PCP, respectively. In both cases, the liver transplant recipient received high‐dose prednisolone for 5 days.

One out of the three had an episode of graft rejection 228 days after PCP (Table 2), of note the immunosuppression was reduced to minimum to reduce the risk of opportunistic infection.

Four out of the seven liver transplant recipients with PCP had chest X‐ray imaging showing bilateral infiltrations compatible with interstitial pneumonia. One patient had a normal chest X‐ray image and CT‐scan, but the patient had clinical symptoms that were compatible with PCP which was confirmed by PCR. Two recipients did not have a chest X‐ray but had chest CT‐scan with lobar infiltration/consolidation. None of the seven patients had a course of the disease which required admission to an ICU and ventilator treatment, and none of them died within 180 days after PCP.

### 3.4 Risk factors for PCP

In uni‐and multivariable models, liver transplant recipients who had at least one episode of rejection had a hazard ratio (HR) for developing PCP of 0.71 ([95% CI: 0.08;6.3], *p* = .76) and 0.76 ([95% CI: 0.09;6.48], *p* = .8). Since there were no cases of PCP in liver transplant recipients while on PCP prophylaxis, we were not able to calculate HR for ongoing PCP prophylaxis.

## 4 DISCUSSION

In this retrospective cohort of liver transplant recipients, we investigated the incidence of PCP in an era with the use of routine PCP prophylaxis. There were no cases of PCP in liver transplant recipients while on PCP prophylaxis. We found the incidence rate of PCP in the first 5 years posttransplantation to be 6.1 per 1000 PYFU. The incidence rate was highest in transplant recipients who did not receive PCP prophylaxis during the first 6 months and the period after discontinuation of routine PCP prophylaxis. We also investigated clinical outcomes and mortality following PCP. All liver transplant recipients who had PCP were admitted to hospital, but none of them were admitted to ICU and none of them died within 180 days after the diagnosis.

Before 2011, PCP prophylaxis was not routinely prescribed to liver transplant recipients at Rigshospitalet. Rostved et al.^9^ reported an outbreak of PCP in 29 SOT recipients, which resulted in a universal prophylaxis program for prevention of PCP. In our cohort, 76% of liver transplant recipients received PCP prophylaxis, and the prophylaxis was generally discontinued 6 months posttransplantation. There was no PCP event in first 6 months posttransplantation in recipients who received PCP prophylaxis. In other words, all events were found the first 6 months in transplant recipients who did not receive prophylaxis or following the 6 month and after discontinuation of PCP prophylaxis. Similar results were reported in a study from Switzerland where the incidence rate of PCP was 3.7 per 1000 PYFU and 11 per 1000 PYFU in SOT recipients who received and did not receive PCP prophylaxis, respectively.^6^ Although, lower incidence rate (0.99 per 1000 PYFU) was reported in a cohort of liver transplant recipients from Spain (ref). Differences between protocols for immune suppression may explain this.^15^

With a median follow‐up of 3.4 years per liver transplant recipients, we found no PCP event after 15th month posttransplantation. This could be due to decrease in immunosuppression. Although, in a recent study from Spain with a median follow‐up of 6.3 years, two out of the five PCP cases were diagnosed after 50th month posttransplantation (late onset PCP).^15^ Similar results were reported from Ireland.^16^ Recommended duration of PCP prophylaxis is 6–12 months posttransplantation.^3^ We showed that six out of seven PJP infections happened after prophylaxis ended. Late onset PCP is a challenge, but considering both risks and benefits of longer prophylaxis, it is difficult to determine the optimal duration of PCP prophylaxis based on current knowledge.^3^ This underlines the need for randomized clinical trials to determine the optimal duration of PCP prophylaxis.

In general, the outcomes of PCP were good. All seven liver transplant recipients who had PCP received antibiotic treatment. None of the patients were admitted to the ICU or needed mechanical ventilation, and none died within 180 days of infections. Other studies have shown higher rates of ICU admission and mortality in SOT recipients with PCP. In the study from Spain, one (20%) out of the five liver transplant recipients with PCP infection was admitted to hospital and one (20%) died.^15^ In the study from Switzerland about 15% of SOT recipients with PCP died during first year after transplantation.^6^ In study from Ireland, five (71%) out of seven PCP patients died.^16^ Fewer patients needing ICU admission and lower mortality in our cohort could be due to the rapid diagnosis and early treatment of PCP. It has been shown that early diagnosis and treatment improves the outcome of PCP.^17^ Moreover, the later posttransplant, the less immunosuppressed recipients, which decreases the risk of a severe PCP. However, the small number of cases of PCP infection in our cohort provides limited power and results should be interpreted with caution.

Two out of seven liver transplant recipients who had PCP had a positive history of rejection within 6 months before the PCP diagnosis, although others have reported that SOT recipients with acute T‐cell‐mediated rejection have 13 times higher chance of PCP infection.^18^ But we could not show this in uni‐ and multivariable Cox proportional hazard models, probably due to low number of cases.

Detailed information about acute graft rejections, antibiotic treatment, and outcomes related to PCP in liver transplant recipients were the strengths of our study. The retrospective design is a limitation, although the information was retrieved from patient records with low risk of bias. In addition, the risk factor analysis is limited by the small cohort of recipients who had PCP. We did not have complete information about types of immunosuppressants, and levels of tacrolimus for all liver transplant recipients. Therefore, we could not look at the relationship of PCP with the use of certain types of immunosuppressants.

In conclusion, we investigated the incidence of PCP in a cohort of liver transplant recipients in an era with the use of routine PCP prophylaxis. We found the incidence rate of PCP in the first 5 years posttransplantation to be 6.1 per 1000 PYFU. There were no cases of PCP in recipients while on prophylaxis. Furthermore, no case with PCP infection was reported after 15th month posttransplantation. This underlines the need for randomized clinical trials to determine the optimal duration of prophylaxis.

## CONFLICT OF INTERESTS

Omid Rezahosseini received a grant from The Research Foundation of Rigshospitalet related, and a grant from A.P. Møller Fonden not related to this study; Andreas D. Knudsen received a grant from The Danish Heart Foundation; Neval E. Warehamwas supported by the Danish National Research Foundation (DNRF) grant no. 126; Susanne D. Nielsen received a grant from Novo Nordisk Foundation and the Independent Research Fund (FSS). Philip B. Andreasen, Dina L. Møller, Magda T. Thomsen, Jenny Knudsen, Jørgen A. L. Kurtzhals, Andreas A. Rostved, Christian R. Pedersen, and Allan Rasmussen have no conflict of interests.

## AUTHOR CONTRIBUTIONS

Philip B. Andreasen, Omid Rezahosseini, Dina L. Møller, Jenny Knudsen, Allan Rasmussen, and Susanne D. Nielsen designed the study. All authors collected the data. Omid Rezahosseini did statistical analyses. Philip B. Andreasen, Omid Rezahosseini, Jenny Knudsen, Allan Rasmussen, and Susanne D. Nielsen wrote the manuscript. Dina L. Møller, Neval E. Wareham, Magda T. Thomsen, Ranya Houmami, Andreas D. Knudsen, Jørgen A. L. Kurtzhals, Allan Rasmussen, and Christian R. Pedersen revised and commented on the manuscript. All authors read and approved the final version of the manuscript.

## Data Availability

The data that support the findings of this study are available from the corresponding author upon reasonable request.
